# RIP3 deficiency attenuated hepatic stellate cell activation and liver fibrosis in schistosomiasis through JNK-cJUN/Egr1 downregulation

**DOI:** 10.1038/s41392-022-01019-6

**Published:** 2022-06-27

**Authors:** Li-Jun Song, Xu-Ren Yin, Sheng-Wen Guan, Hong Gao, Pan-Pan Dong, Cong-Jin Mei, Ying-Ying Yang, Ying Zhang, Chuan-Xin Yu, Zi-Chun Hua

**Affiliations:** 1 School of Life Sciences and the State Key Laboratory of Pharmaceutical Biotechnology, Nanjing University, Nanjing, PR China; 2 Key Laboratory of National Health and Family Planning Commission on Parasitic Disease Control and Prevention, Jiangsu Provincial Key Laboratory on Parasite and Vector Control Technology, Jiangsu Institute of Parasitic Diseases, Wuxi, PR China; 3 Public Health Research Center at Jiangnan University, Wuxi, PR China; 4 Department of Pathology, Nanjing Drum Tower Hospital, The Affiliated Hospital of Nanjing University Medical School, Nanjing, PR China; 5 School of Biopharmacy, China Pharmaceutical University, Nanjing, PR China; 6 Changzhou High-Tech Research Institute of Nanjing University and Jiangsu TargetPharma Laboratories Inc, Changzhou, PR China

**Keywords:** Infectious diseases, Target identification

**Dear Editor**,

Activated hepatic stellate cells (HSCs) synthesizing a large amount of extracellular matrix (ECM) are the key events to hepatic fibrosis.^1^ However, there are no effective drugs for curing liver fibrosis in the clinic.^1^ Therefore, clarifying the formation mechanism of liver fibrosis is of great importance for finding anti-liver fibrosis drug targets. Recently, an increasing amount of research has shown that necroptosis regulated by receptor-interacting protein kinase 3 (RIP3) plays an important role in inflammatory disease injury and could be used as a drug target.^2^

We found that RIP3 was highly expressed in liver tissues of wild-type (WT) mice infected with *Schistosoma japonicum* (*S. japonicum*), mainly at 6 w (weeks) and 8 w post-infection (Fig. 1a), which was consistent with the liver pathological damage of schistosomiasis (Fig. 1e, f), and was mainly expressed in hepatocytes and CD45^+^ cells rather than HSCs (Fig. 1b). Therefore, RIP3 could be a drug target of pathogenic liver fibrosis, which remains poorly understood.

**Fig. 1:**
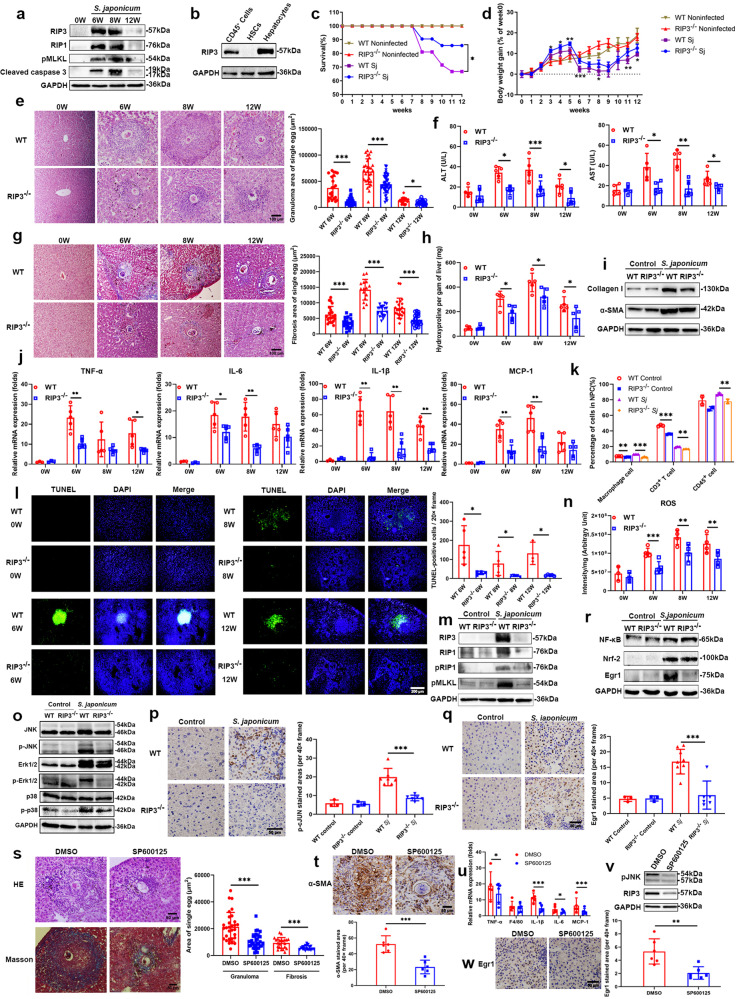
Role of RIP3 in liver fibrosis induced by schistosomiasis. **a** Western blot detection of RIP3, RIP1, pMLKL and cleaved caspase 3 expression in the liver. **b** Western blot detection of RIP3 expression in CD45^+^ cells, HSCs, and hepatocytes. **c** Comparison of the survival rates of WT and RIP3^−/−^ mice before and after schistosome infection (*n* = 5). **d** Weight gain percentage of WT and RIP3^−/−^ mice before and after schistosome infection (*n* = 5). **e** Egg granuloma in infected WT and RIP3^−/−^ mouse livers at 0 w, 6 w, 8 w, and 12 w (HE staining, scale = 100 μm). **f** Serum ALT and AST levels of infected WT and RIP3^−/−^ mice at 0 w, 6 w, 8 w, and 12 w (*n* = 5). **g** Degree of liver fibrosis in infected WT and RIP3^−/−^ mice at 0 w, 6 w, 8 w, and 12 w (Masson staining, scale = 100 μm). Collagen was dyed blue. **h** Hydroxyproline levels in WT and RIP3^−/−^ mouse liver tissues at 0 w, 6 w, and 8 w (*n* = 5). **i** Collagen I and α-SMA expression levels in HSCs from infected or noninfected (control) mice detected by Western blot analysis. **j** The mRNA expression levels of TNF-α, IL-6, IL-1β, and MCP-1 in infected WT and RIP3^−/−^ mouse livers at 0 w, 6 w, 8 w, and 12 w detected by fluorescence quantitative PCR (*n* = 5). **k** Flow cytometry detection of the ratio of macrophages, CD3^+^ T cells, and CD45^+^ cells in mouse livers before and after infection. **l** TUNEL staining of infected mouse livers at 0 w, 6 w, 8 w, and 12 w. Dead cells were dyed green. Nuclei were stained with DAPI (blue) (scale = 200 μm). **m** RIP3, RIP1, pRIP1, and pMLKL expression levels in infected mouse livers. **n** ROS levels in infected WT and RIP3^−/−^ mouse livers at 0 w, 6 w, 8 w, and 12 w (*n* = 5). **o** Western blot detection of JNK, Erk1/2, P38, and phosphorylated protein expression in WT and RIP3^−/−^ mouse livers before and 8 w after infection. **p** Immunohistochemistry detection of p-cJUN expression (brown) in WT and RIP3^−/−^ mouse livers before and after infection with *S. japonicum* (scale = 50 μm). Nuclei were stained with hematoxylin (blue). **q** Immunohistochemistry detection of Egr1 expression (brown) in WT and RIP3^−/−^ mouse livers before and after infection with *S. japonicum* (scale = 50 μm). Nuclei were stained with hematoxylin (blue). **r** Expression of NF-κB, Nrf-2 and Egr1 in WT and RIP3^−/−^ mouse livers before and 8 weeks after infection, as detected by Western blot analysis. **s** Egg granuloma (HE staining, scale = 50 μm) and fibrosis (Masson staining, scale = 50 μm) in SP600125-treated infected mice (*n* = 5). Collagen was dyed blue by Masson staining. **t** Immunohistochemistry detection of α-SMA expression (brown) in the livers of SP600125-treated infected mice. Nuclei were stained with hematoxylin (blue) (scale = 50 μm). **u** TNF-α, F4/80, IL-1β, IL-6, and MCP-1 mRNA levels in the livers of SP600125-treated infected mice, as detected by fluorescence quantitative PCR (*n* = 5). **v** RIP3 expression in the livers of SP600125-treated infected mice, as detected by Western blot analysis. **w** Immunohistochemistry detection of Egr1 (brown) expression in liver (scale = 50 μm). Nuclei were stained with hematoxylin (blue). Experiments were repeated ≥3 times, and all the data are shown as the mean ± SD, *P < 0.05; **P < 0.01; ***P < 0.001

Subsequently, WT and RIP3 knockout mice were used for infection. The results showed that RIP3 knockout improved the survival rate and body weight of the infected mice (Fig. 1c, d). HE staining showed that RIP3 deficiency reduced the areas of egg granuloma in the infected mouse livers at 6 w, 8 w, and 12 w post-infection (Fig. 1e), with significantly decreased ALT and AST levels (Fig. 1f). Masson staining showed that RIP3 deficiency reduced the areas of egg fibrosis at 6 w, 8 w, and 12 w post-infection (Fig. 1g). Compared with WT mice, RIP3^−/−^ mice had lower levels of hydroxyproline (Fig. 1h), lower mRNA levels of collagen I, collagen III, α-smooth muscle actin (α-SMA), and tissue inhibitor of metalloproteinases (TIMP1), and higher mRNA levels of matrix metalloproteinases (MMP9) (Supplementary Fig. S1c–g), reflecting significantly reduced collagen in the liver. HSCs were isolated and purified from mouse livers before and 8 w after infection. RIP3^−/−^ mice had significantly lower expression of collagen I and α-SMA in HSCs (Fig. 1i), suggesting that RIP3 deficiency reduced HSC activation induced by schistosome infection. The above results indicated that RIP3 deficiency alleviated the pathological liver damage caused by *S. japonicum* infection.

Since RIP3 is important in inflammatory disease injury,^2^ we detected the mRNA expression levels of the inflammatory factors including tumor necrosis factor (TNF-α), interleukin-1β (IL-1β), and IL-6 and the monocyte chemoattractant protein (MCP-1) in the infected mouse livers, the percentage of macrophages, CD3^+^ T cells, and CD45^+^ monocytes and the expression of F4/80 in liver tissue. The results showed that RIP3 gene knockout reduced the above indicators (Fig. 1j, k, Supplementary Fig. S2), indicating that RIP3 deficiency reduced the degree of inflammation in the livers of mice infected with *S. japonicum*.

We further determined the type of cell death using TUNEL and detecting the expression of the necroptosis marker proteins pMLKL and RIP1, the residues of cytokeratin 18 (CK18) and cleaved caspase 3 from apoptosis, LC3A/B for autophagy, the level of ATP for necrosis, the content GSH and the mRNA expression of glutathione peroxidase 4 (GPX4) and acyl-CoA synthetase long chain family member 4 (ACSL4) for ferroptosis in the liver. RIP3 knockout decreased the number of dead cells in mouse liver tissues at 6 w, 8 w, and 12 w (Fig. 1l), the expression of the key proteins pMLKL and pRIP1 (Fig. 1m), the reactive oxygen species (ROS) levels of necroptosis (Fig. 1n), the residues of CK18 and cleaved caspase 3 resulting from apoptosis, and also the level of ATP in the liver, but had little effect on autophagy or ferroptosis after infection (Supplementary Fig. S3), confirming the key role of RIP3 in not only cell necroptosis but also apoptosis, rather than autophagy and ferroptosis caused by *S. japonicum*, which was different from that RIP3 deficiency decreased necroptosis but promoted apoptosis in the liver in response to HFD, leading to accelerated fibrosis.^3^

Furthermore, we detected downstream signaling pathways of RIP3. RIP3 deficiency downregulated the phosphorylation of the JNK and Erk1/2 proteins of the MAPK family (Fig. 1o) and the expression of the transcription factor p-cJUN and Egr1 (Fig. 1p, q), but it did not regulate NF-κB and Nrf-2 (Fig. 1r) in schistosomiasis-induced liver injury differently from the other disease models,^4,5^ suggesting that the proteins downstream of RIP3 may be related to the initial damage factors and pathological microenvironment.

Furthermore, to explore how hepatocyte death induced by RIP3 leads to the activation of hepatic stellate cells, the human hepatocyte lines L-02 or HepG2 was cocultured with the hepatic stellate cell line LX-2. Knockdown or overexpression of RIP3 in L-02 or HepG2 cells (Supplementary Fig. S4a, d and Supplementary Fig. S5a, c) reduced or increased necroptosis (Supplementary Fig. S4b, e and Supplementary Fig. S5a, c), apoptosis (Supplementary Fig. S4g, h and Supplementary Fig. S5e, f), downregulated or upregulated collagen I and α-SMA expression in LX-2 cells (Supplementary Fig. S4c, f and Supplementary Fig. S5b, d), and decreased or increased LX-2 cell activation, respectively. The levels of TNF-α (Supplementary Fig. S4j, l and Supplementary Fig. S5h, j) and ROS (Supplementary Fig. S4i, k and Supplementary Fig. S5g, i) in the medium fell or rose accordingly, suggesting that RIP3 might regulate inflammatory factors and ROS production to activate HSCs. In L-02 or HepG2 cell, we further confirmed the downstream signaling molecule JNK of RIP3, but has less effect on Erk1/2 and the phosphorylation (Supplementary Fig. S6a–h). And RIP3–ROS–JNK–ROS was a positive feedback regulatory process at the cellular level (Supplementary Fig. S6i–l).

Finally, to verify the role of JNK on RIP3 in liver fibrosis in schistosomiasis and the relationship between JNK and RIP3, we treated infected mice with 50 mg/kg SP600125 intraperitoneally, the inhibitor of JNK, for 4 weeks. The results showed that SP600125 reduced egg granuloma and fibrosis areas (Fig. 1s), the expression of α-SMA (Fig. 1t), the level of hydroxyproline (Supplementary Fig. S7a), the mRNA levels of inflammatory and fibrosis-related factors (Fig. 1u, Supplementary Fig. S7b), the proportion of inflammation-related cells (Supplementary Fig. S7c) and the ROS level (Supplementary Fig. S7d). In addition, the JNK inhibitor also downregulated RIP3 expression (Fig. 1v), further confirming that RIP3–JNK was positively feedback regulated. JNK inhibitors also downregulated the expression of Egr1 (Fig. 1w), which suggested that Egr1 was a downstream transcription factor of JNK. The detection of transcription factor fluorescent reporter gene showed that Egr1 could regulate the expression of TNF-α (Supplementary Fig. S8).

In conclusion, the present study demonstrated that RIP3 deficiency attenuated liver fibrosis induced by *S. japonicum*. RIP3–JNK–cJUN/Egr1 axis positive feedback regulated inflammatory factor and ROS production, which activated HSCs, promoting schistosomiasis liver fibrosis. Inhibiting JNK prevented schistosomiasis liver fibrosis. Thus RIP3 and its signal pathway could be novel drug targets for pathogenic liver fibrosis (Supplementary Fig. S9).

## Supplementary information

Supplementary Materials

## Data Availability

All data generated or analyzed during this study are included either in this article or in the supplementary information files.
