# Supplementary material for: RIP3 deficiency attenuated hepatic stellate cell activation and liver fibrosis in schistosomiasis through JNK-cJUN/Egr1 downregulation
Source: Signal Transduct Target Ther. 2022 Jun 27;7:193. doi: 10.1038/s41392-022-01019-6 (PMC9234041; doi:10.1038/s41392-022-01019-6)
Supplement: Supplementary file 1 — Supplementary Materials [file 41392_2022_1019_MOESM1_ESM.docx]

Supplementary Materials for

RIP3 deficiency attenuated hepatic stellate cell activation and liver fibrosis in schistosomiasis through JNK-cJUN/Egr1 downregulation

Li-Jun Song^1,2,3^, Xu-Ren Yin^2^, Sheng-Wen Guan^1^, Hong Gao^4^, Pan-Pan Dong^2^, Cong-Jin Mei^2^, Ying-Ying Yang^2^, Ying Zhang^2^, Chuan-Xin Yu^2^, Zi-Chun Hua^1,5,6*^

^1^School of Life Sciences and the State Key Laboratory of Pharmaceutical Biotechnology, Nanjing University, Nanjing, PR China

^2^Key Laboratory of National Health and Family Planning Commission on Parasitic Disease Control and Prevention, Jiangsu Provincial Key Laboratory on Parasite and Vector Control Technology, Jiangsu Institute of Parasitic Diseases, Wuxi, PR China

^3^Public Health Research Center at Jiangnan University, Wuxi, PR China

^4^Department of Pathology, Nanjing Drum Tower Hospital, The Affiliated Hospital of Nanjing University Medical School, Nanjing, PR China

^5^School of Biopharmacy, China Pharmaceutical University, Nanjing, PR China

^6^Changzhou High-Tech Research Institute of Nanjing University and Jiangsu TargetPharma Laboratories Inc., Changzhou, PR China

* Correspondence:

Corresponding Author Zi-Chun Hua; [huazc@nju.edu.cn](mailto:huazc@nju.edu.cn)

**This PDF file includes:**

Materials and Methods

Supplementary Figure S1 to S9

Supplementary Table S1 to S2

**Materials and Methods**

**Animals, parasites, and cells**

RIP3^-/-^ mice were generously gifted by Professor Wang Xiaodong of the Beijing Institute of Life Sciences and were raised in the Department of Experimental Animals, Jiangsu Institute of Parasitic Diseases. WT C57BL/6 mice (6–8 weeks old) were purchased from the Yangzhou University Medical Animal Center. All mice were housed in the facility and given food and water according to the Convention. The mice were infected and then killed (carbon dioxide asphyxia) in accordance with the Ministry of Science and Technology of the People’s Republic of China Guidelines for feeding and use of experimental animals ((2006) No. 398); we also minimized the animals’ suffering in accordance with the guidelines.

*S. japonicum* cercariae (Jiangsu strain) were hatched from infected snails provided by the Department of Snail Biology, Jiangsu Institute of Parasitic Diseases. The L-02 normal human hepatocyte line was provided by Xiamen Antithela Biotechnology (Xiamen, China). The HepG2 cells line was purchased from the Shanghai Institute of Biochemistry and Cell Biology of the Chinese Academy of Sciences (Shanghai, China). The LX-2 HSCs and HEK-293 cells line is maintained in our laboratory. All cells were cultured in Dulbecco’s modified Eagle’s medium (DMEM) containing 10% fetal bovine serum.

**Establishment of an *S. japonicum*-induced liver fibrosis model**

WT and RIP3^-/-^ mice, 5 mice in each group, were infected with *S. japonicum* cercariae (12 ± 1). The mice were sacrificed at 6 weeks (w), 8 w, and 12 w after infection. The adult worms were collected, and the livers were digested in 5% KOH. The numbers of eggs in the liver were counted three times. Uninfected mice of the same age were used as the control, and the mice were weighed every week.

**Serum collection and alanine aminotransferase (ALT) and aspartate aminotransferase (AST) detection in mice**

Mouse blood was collected from the eyeballs and centrifuged at 1000 × g for 5 min, and the serum was stored at -80°C. Serum ALT an AST levels were detected using a kit (Nanjing Jiancheng Biotechnology, Nanjing, China).

**Determination of hydroxyproline in mouse liver tissue**

The hydroxyproline level in 30–100 mg mouse liver was detected by a hydroxyproline assay kit (Nanjing Jiancheng Biotechnology).

**Determination of ATP in mouse liver tissue**

The ATP level in 100 mg mouse liver was detected by an ATP detection assay kit (Beijing Solarbio Technology, Beijing, China).

**GSH content in mouse liver tissue**

The GSH content in 100 mg mouse liver was detected by a GSH detection assay kit (Beijing Solarbio Technology, Beijing, China).

**Hematoxylin–eosin (HE) and Masson staining staining of mouse liver tissue**

The right anterior lobe of the mouse liver was fixed in 4% paraformaldehyde. The tissue was dehydrated and cleared successively in ethanol and xylene. The mouse livers of *S. japonicum*-induced liver fibrosis were stained with hematoxylin–eosin (HE) and Masson staining kits (Beijing Solarbio Technology, Beijing, China). We randomly viewed 6–8 fields of vision from each section under the microscope (Olympus, Japan) and photographed them to analyze the area of granuloma and fibrosis of a single egg using cellSens Dimension (Olympus, Japan).

**Fluorescence quantitative PCR**

Total RNA was extracted from mouse livers using TRIzol. RNA (500 μg) was reverse-transcribed into cDNA by a reverse transcription kit (Roche, Basel, Switzerland). cDNA was used as a template for each cytokine mRNA level using SYBR Green I Master Mix (Roche) and a LightCycler 480 instrument (Roche). The reaction procedure was as follows: 95°C for 30 s; 40 cycles of 95°C for 5 s, 56°C for 10 s, 72°C for 15 s; 95°C for 15 s, 60°C for 10 s. 18S was used as the internal control, and the fold changes were quantified by the comparative threshold cycle (2^-△△Ct^) method. The primer sequences are shown in Table S1, and all primer sequences were blasted in the National Center for Biotechnology Information (NCBI) to ensure their specificities.

**Western blotting**

Mouse liver tissue was lysed with radioimmunoprecipitation assay (RIPA) lysate (Cwbiotech, Nanjing, China), and the supernatant was centrifuged at 12 000 ×g. The protein concentration was detected by the bicinchoninic acid (BCA) method. Protein (50–100 μg) was electrophoresed using 12% sodium dodecyl sulfate–polyacrylamide gel electrophoresis (SDS–PAGE) and then transferred to polyvinylidene fluoride (PVDF) membranes (Millipore, Massachusetts, USA) by a semidry transfer instrument (Bio–Rad, California, USA). The membranes were blocked with 5% skimmed milk powder at 37°C for 1 h and incubated overnight at 4°C with primary antibodies (α-SMA, collagen I, RIP3, RIP1, JNK, Erk1/2, p38, phosphorylated (p) JNK, pErk, p-p38, pRIP1, pMLKL, NF-κB, Nrf-2, Egr1, LC3A/B, cleaved caspase 3, Flag, GAPDH [all, 1:1000], Cell Signaling Technology, Massachusetts, USA). Then, the membranes were incubated for 1 h at room temperature with goat anti-mouse or anti-rabbit secondary antibody (Jackson, Pennsylvania, USA) and developed with electrochemiluminescence (ECL) (Vazyme, Nanjing, USA). GAPDH or HSP90 was used as the housekeeping gene. The membranes were scanned with the GelDoc XR+ system (Bio-Rad, USA).

**Immunohistochemistry**

Pathological liver sections (4 μm) were dewaxed in water; sodium citrate repair solution was used for antigen repair by microwave. Then, 10% goat serum was used for antigen blocking at room temperature for 1 h. The sections were incubated overnight at 4°C with primary antibodies (α-SMA, p-cJUN, Egr1, F4/80^+^, [all, 1:200], Cell Signaling Technology, Massachusetts, USA; Cytokeratin 18 (CK18), 1:200, Roche, Basilea, Switzerland). Then, the sections were incubated with horseradish peroxidase-labeled secondary antibody at room temperature for 1 h. The sections were developed with DAB reagent solution, and cores were dyed with hematoxylin solution. Then, the sections were dehydrated, transparent, and sealed with neutral resin. We randomly viewed 3–4 fields of vision from each section under the microscope (Olympus, Japan) and the stained positive areas were analysed by Image Pro Plus software (Media Cybernetics, Houston, USA).

**TUNEL assay**

Pathological liver sections (4 μm) were dewaxed in water; TUNEL (terminal deoxynucleotidyl transferase dUTP nick end labeling)-positive cells were detected with a TUNEL kit (Vazyme Biotech, Nanjing, China). 3–4 fields of vision from each section were viewed under the microscope (Olympus, Japan) and the stained positive cells were counted by Image Pro Plus software (Media Cybernetics, Houston, USA).

**Flow cytometry**

Mouse liver was cut into small pieces, and collagenase and DNase were added and digested at 37°C for 30 min. Then, the liver was passed through 200- and 400-mesh sieves and centrifuged at 300 × g for 5 min, and the digestion was stopped with phosphate-buffered saline (PBS). After 4 min of centrifugation at 50 × g and 25 × g, the liver parenchymal cells were removed. The supernatant was transferred to a new centrifuge tube and centrifuged at 300 × g for 5 min, and the cells were counted. 2 × 10^6^ cells were added by the following labeled antibodies: CD11b-FITC and F4/80-PE (for macrophages); CD3-PE (for T cells); and CD45-FITC (for monocytes) (all, BD, Franklin Lakes, NJ, USA) and detected by FACSVerse Flow Cytometer (BD, Franklin Lakes, NJ, USA) and analyzed with FlowJo software (Tree Star, Ashland, OR, USA). The apoptosis of L-02 or HepG2 cell was stained used an apoptosis kit (BD, Franklin Lakes, NJ, USA), detected by FACSVerse Flow Cytometer (BD, Franklin Lakes, NJ, USA) and analyzed with FlowJo software (Tree Star, Ashland, OR, USA).

**Cell culture and detection**

RIP3 small interfering RNA (siRNA) was synthesized by Suzhou Gemma Biological Gene (Suzhou, China). Table S2 lists the siRNA sequences.

The RIP3 gene was synthesized and cloned into the pCMV-N-Flag vector by Nanjing GenScript Biotechnology (Nanjing, China). L-02 or HepG2 cells (1.5 × 10^5^) were transferred to 6-well plates and cultured overnight. The cells were transfected with siRNA using Vazyme Biotech transfection reagent and with RIP3/pCMV-N-Flag vector using Attractene Transfection Reagent (Qiagen, Germantown, MD, USA).

On the second day, LX-2 cells were cocultured with L-02 or HepG2 cells in a nested membrane culture plate (Corning, New York, USA), and 50 μg/mL SEA was added for 48 h. pMLKL expression in L-02, Collagen I and α-SMA protein expression in LX-2 cells was detected by western blotting. Reactive oxygen species (ROS) levels in L-02 or HepG2 cells were detected using a DCFH-DA kit (BioLegend, San Diego, CA, USA), and TNF-α in the cell culture supernatant were detected using an ELISA kit (Multisciences, Hangzhou, China).

**Immunofluorescence**

LX-2 cells cocultured with L-02 cells were used for cell climbing. After climbing, the cells were fixed in 4% paraformaldehyde. Then, the cells were blocked with 10% goat serum, incubated with antibody against α-SMA (1:200, Cell Signaling Technology, Massachusetts, USA) overnight at 4°C, and then incubated with the fluorescent secondary antibody at room temperature for 1 h. The samples were sealed with neutral resin containing anti-fluorescence quenching agent and observed immediately under a fluorescence microscope (Olympus, Japan), and 3–4 fields of vision from each section were photographed.

**SP600125 treatment of infected mice**

WT mice (5 mice in each group) were infected abdominally with 12 ± 1 S. japonicum cercariae. Four weeks after infection, each mouse was intraperitoneally injected with 50 mg/kg SP600125 once a day. After 4 weeks of continuous administration, the mice were sacrificed after 8 weeks of infection. The number of *S. japonicum* adults and eggs were counted. The areas of granuloma and fibrosis of single eggs were detected by HE and Masson staining. Liver tissue was obtained for immunohistochemistry to detect α-SMA and Egr1 expression; serum ALT and AST levels were detected with ALT and AST test kits (Jiancheng Bioengineering Institute, Nanjing, China); hydroxyproline levels were detected with a hydroxyproline kit (Jiancheng Bioengineering Institute, Nanjing, China). Collagen I, collagen III, α-SMA, MMP9, TIMP1, TNF-α, F4/80, IL-1β, IL-6, and MCP-1 (monocyte chemoattractant protein-1) mRNA levels were detected with real-time PCR, and the ratio of CD3^+^ cells, CD45^+^ cells, and macrophages was detected by flow cytometry. ROS levels and RIP3 expression in the liver tissue were detected using an ROS detection kit (Bestbio, Shanghai) and western blotting, respectively.

**Detection of transcription factor fluorescent reporter gene**

The possible transcription factor binding sites in the promoter region was analyzed and predicted in http://jaspar.binf.ku.dk/. The transcriptional regulation of its promoter was detected by double luciferase reporter gene. The target promoter fragment sequence was synthesized and inserted into luciferase reporter gene plasmid (pGL3 basic) by Genomeditech (Shanghai, China). Reporter gene plasmid and transcription factor expression plasmid were co-transfected into HEK-293 cells. The protein was extracted and luciferase was detected used a Luciferase reporter gene detection kit (Genomeditech, Shanghai, China). The substrate was added to determine the activity of luciferase. The relative fluorescence intensity was tested by using multifunctional enzyme labeling instrument (infinite M1000, Tecan, Switzerland).

**Statistical analysis**

The percentage of weight gain, blood ALT and AST levels, liver tissue hydroxyproline levels, mRNA levels of inflammatory factors, and the proportion of inflammation-related cells in the liver tissue between the two groups were tested by t test. SPSS 13.0 (IBM, Armonk, NY, USA) was used for the statistical analyses. Differences between mean values were considered significant at *P* < 0.05.


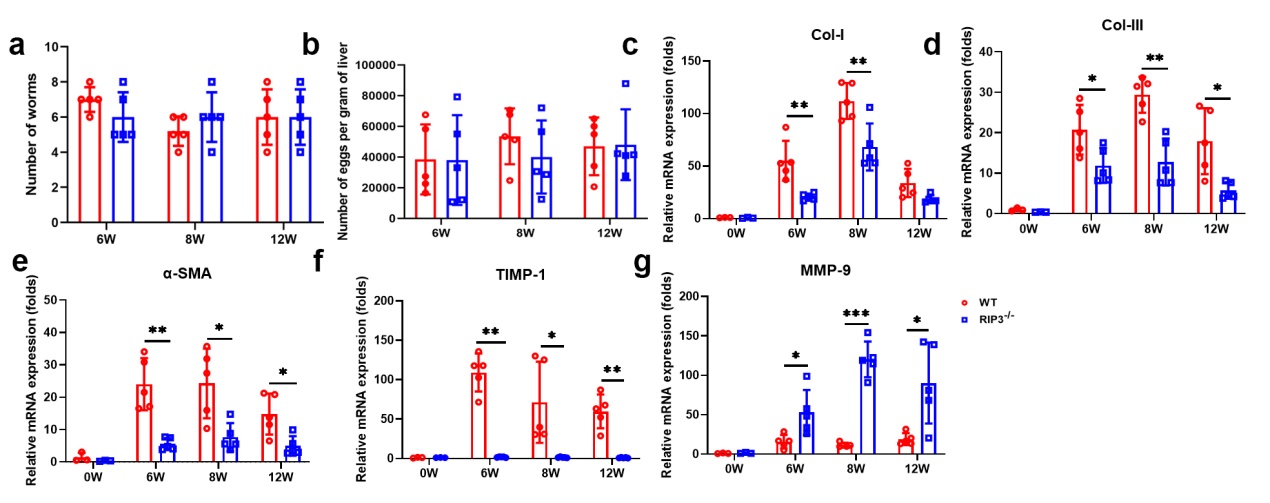


**Supplementary Fig. S1 Infection burdens and mRNA expression levels of fibrosis factors in WT and RIP3^-/-^ mouse liver tissues. a.** The adult worm burden of WT and RIP3^-/-^ mice at 6 w, 8 w, and 12 w post infection (n = 5). **b.** The egg burden of WT and RIP3^-/-^ mice at 6 w, 8 w, and 12 w after infection (n = 5). **c-g.** mRNA expression levels of collagen I (c), collagen III (d), α-SMA (e), TIMP1 (f), and MMP9 (g) in WT and RIP3^-/-^ mouse liver tissues at 0 w, 6 w, 8 w, and 12 w detected by fluorescence quantitative PCR (n = 5) (**P* < 0.05, ***P* < 0.01, ****P* < 0.001).


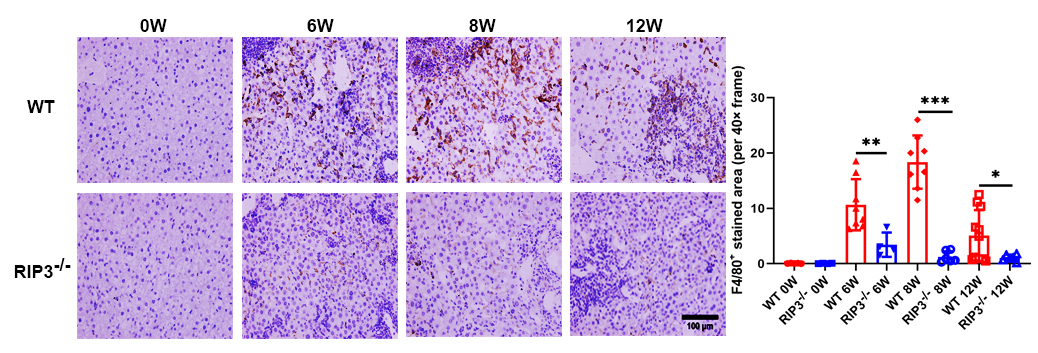


**Supplementary Fig. S2** **Immunohistochemistry detection of F4/80^+^ expression (brown) levels in WT and RIP3^-/-^ mouse livers at 0 w, 6 w, 8 w and 12 w.** Nucleus was stained with hematoxylin (blue). Scale = 100 μm (**P* < 0.05, ***P* < 0.01, ****P* < 0.001).


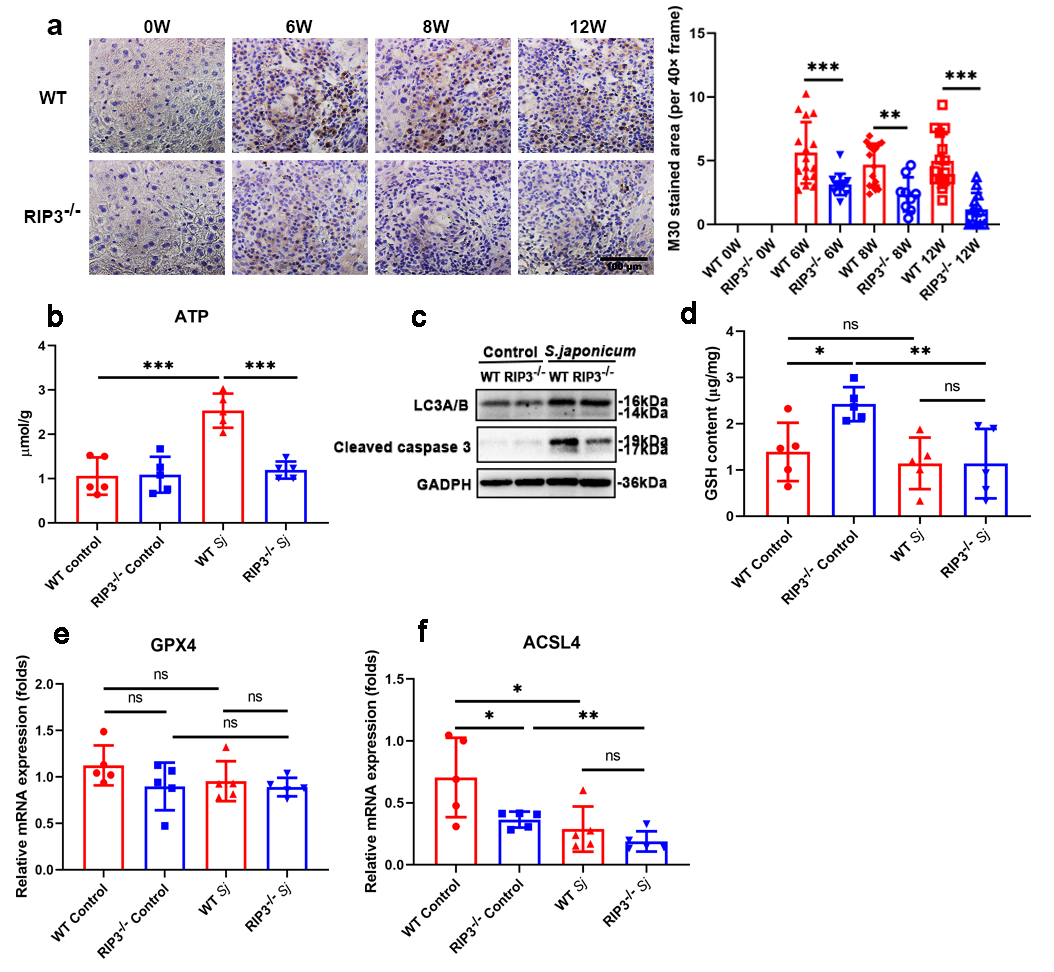


**Supplementary Fig. S3 Cell death in WT and RIP3^-/-^ mouse livers. a.** Immunohistochemistry detection of Cytokeratin 18 (CK18) expression (brown) in infected WT and RIP3^-/-^ mouse livers at 0 w, 6 w, 8 w and 12 w (scale = 100 μm). Nucleus was stained with hematoxylin (blue). **b.** The level of ATP in the liver of WT and RIP3^-/-^ mice before and at 8 w after infection. **c.** The expression level of LC3A/B of autophagy, cleaved of caspase 3 of apoptosis in the liver of WT and RIP3^-/-^ mice before and at 8 w after infection. **d.** The GSH content in the liver of WT and RIP3^-/-^ mice before and at 8 w after infection. **e.** The relative mRNA expression of GPX4 in the liver of WT and RIP3^-/-^ mice before and at 8 w after infection. **f.** The relative mRNA expression of ACSL4 in the liver of WT and RIP3^-/-^ mice before and at 8 w after infection. (**P* < 0.05, ***P* < 0.01, ****P* < 0.001).


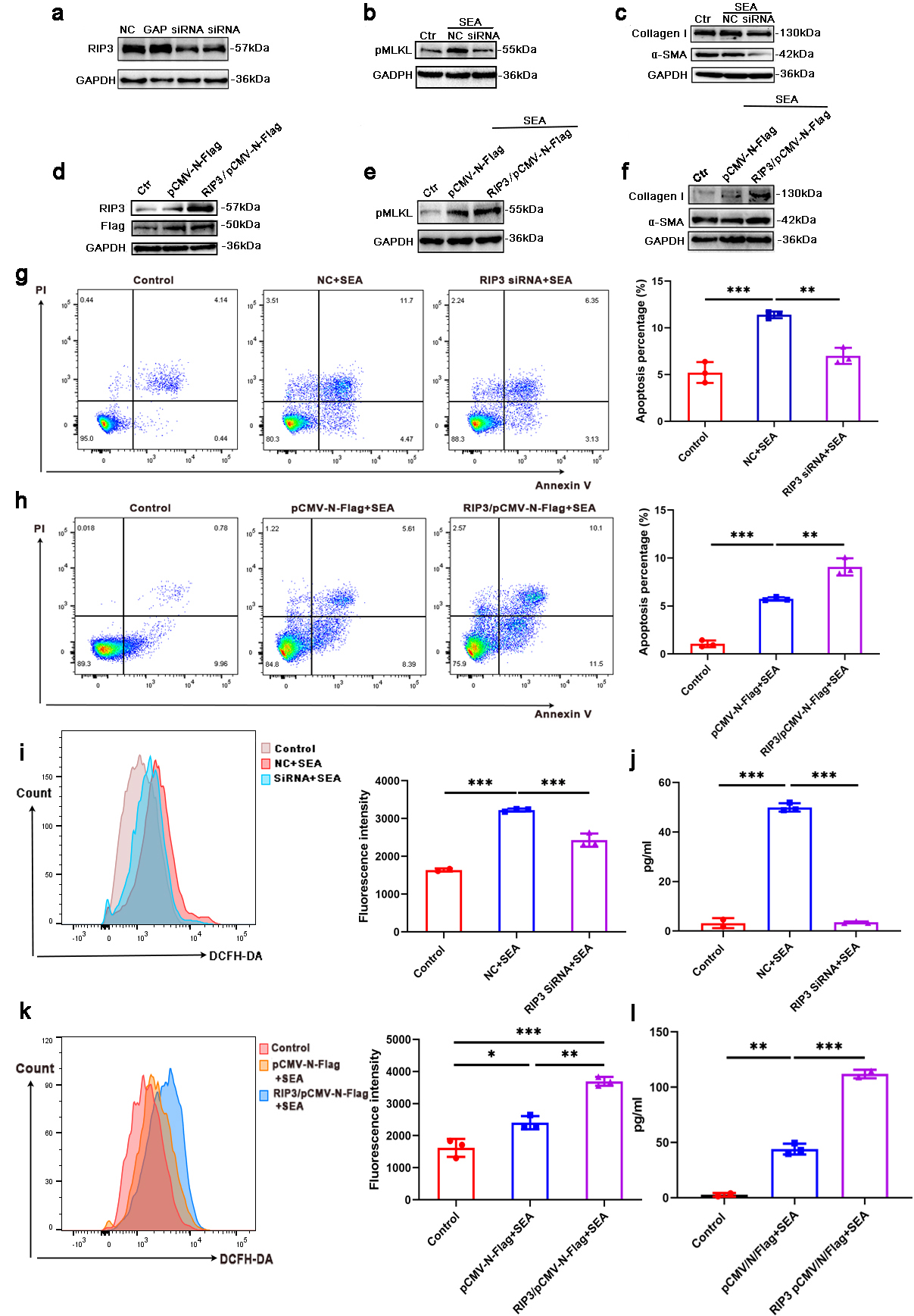


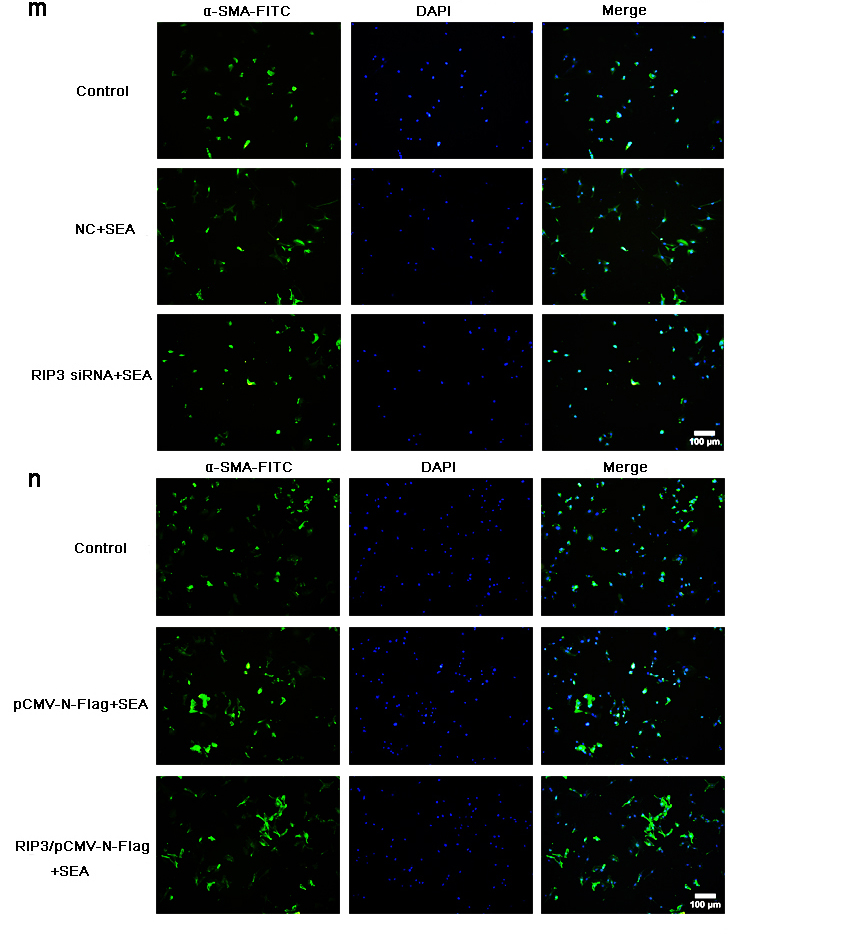


**Supplementary Fig. S4 Effect of knockdown or overexpression of RIP3 in L-02 on LX-2 activation. a.** The expression level of RIP3 in L-02 cells transfected with RIP3 siRNA. **b.** The expression level of pMLKL in L-02 cells transfected with RIP3 siRNA. **c.** The expression level of collagen I and α-SMA in LX-2 cells co-cultured with L-02 transfected with RIP3 siRNA. **d.** The expression level of RIP3 in L-02 cells after transfection with the RIP3 overexpression plasmid. **e.** The expression level of pMLKL in L-02 cells overexpressing RIP3. **f.** The effect of L-02 cells overexpressing RIP3 on collagen I and α-SMA expression in LX-2 cells. **g.** Flow cytometry detection and statistical analysis of cell apoptosis in L-02 cells transfected with RIP3 siRNA. **h.** Flow cytometry detection and statistical analysis of cell apoptosis in L-02 cells transfected with RIP3 overexpression plasmid. **i.** Flow cytometry detection and statistical analysis of ROS fluorescence intensity in L-02 cells transfected with RIP3 siRNA. **j.** Effect of RIP3 knockdown on TNF-α expression levels in L-02 hepatocytes. **k.** Flow cytometry detection and statistical analysis of ROS fluorescence intensity in L-02 cells transfected with RIP3 overexpression plasmid. **l.** Effect of RIP3 overexpression on TNF-α secretion by L-02 cells. **m.** Immunofluorescence detection of α-SMA expression in LX-2 cells cocultured with L-02 cells transfected with RIP3 siRNA (scale = 100 μm). **n.** Immunofluorescence detection of α-SMA expression in LX-2 cells cocultured with L-02 cells transfected with RIP3 overexpression plasmid (scale = 100 μm) (**P* < 0.05, ***P* < 0.01, ****P* < 0.001).


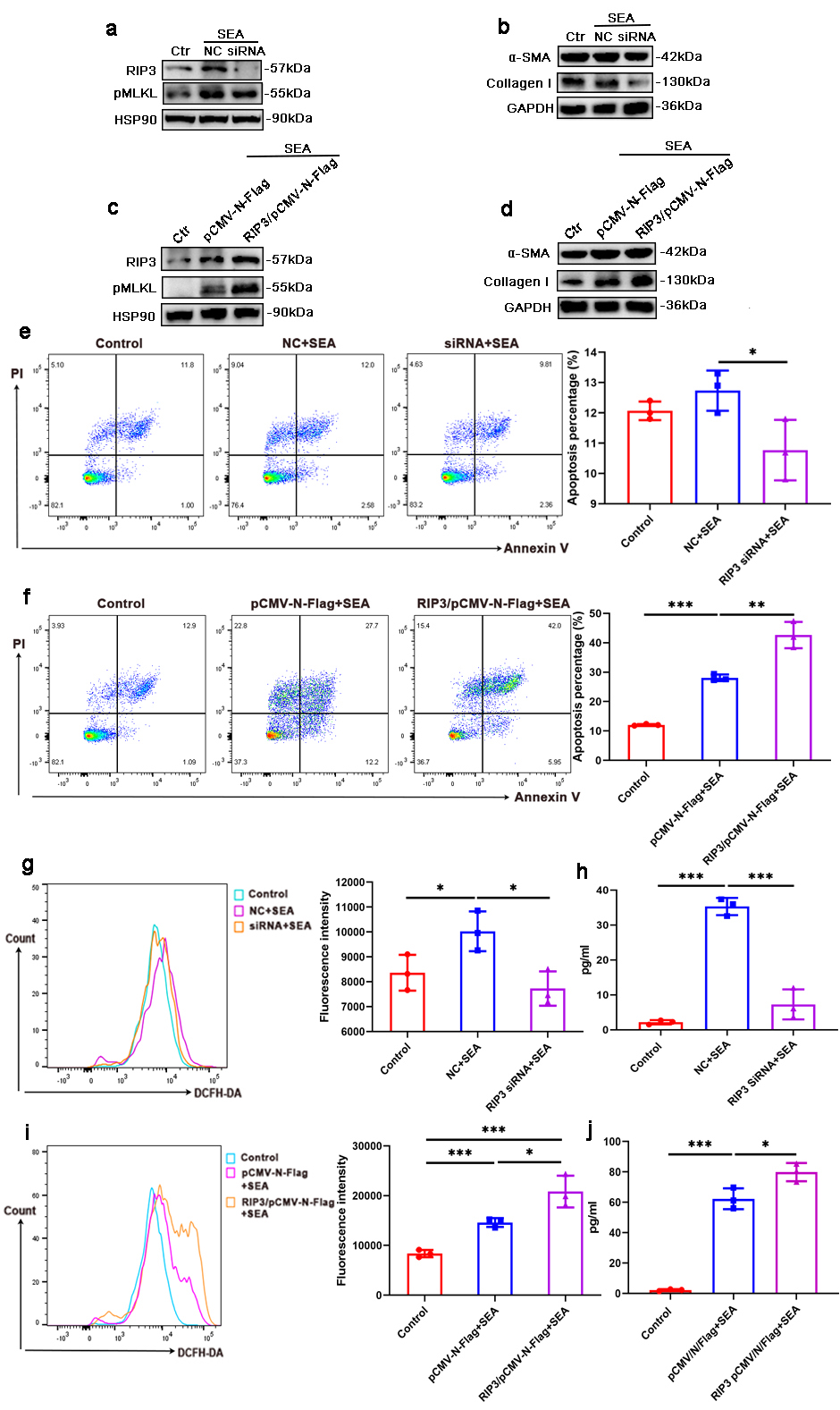


**Supplementary Fig. S5 Effect of knockdown or overexpression of RIP3 in HepG2 on LX-2 activation. a.** The expression level of RIP3 and pMLKL in HepG2 cells transfected with RIP3 siRNA. **b.** The expression level of collagen I and α-SMA in LX-2 cells co-cultured with L-02 transfected with RIP3 siRNA. **c.** The expression level of RIP3 and pMLKL in HepG2 cells after transfection with the RIP3 overexpression plasmid. **d.** The effect of L-02 cells overexpressing RIP3 on collagen I and α-SMA expression in LX-2 cells. **e.** Flow cytometry detection and statistical analysis of cell apoptosis in HepG2 cells transfected with RIP3 siRNA. **f.** Flow cytometry detection and statistical analysis of cell apoptosis in HepG2 cells transfected with RIP3 overexpression plasmid. **g.** Flow cytometry detection and statistical analysis of ROS fluorescence intensity in HepG2 cells transfected with RIP3 siRNA. **h.** Effect of RIP3 knockdown on TNF-α expression levels in HepG2 hepatocytes. **i.** Flow cytometry detection and statistical analysis of ROS fluorescence intensity in HepG2 cells transfected with RIP3 overexpression plasmid. **j.** Effect of RIP3 overexpression on TNF-α secretion by HepG2 cells (**P* < 0.05, ***P* < 0.01, ****P* < 0.001).


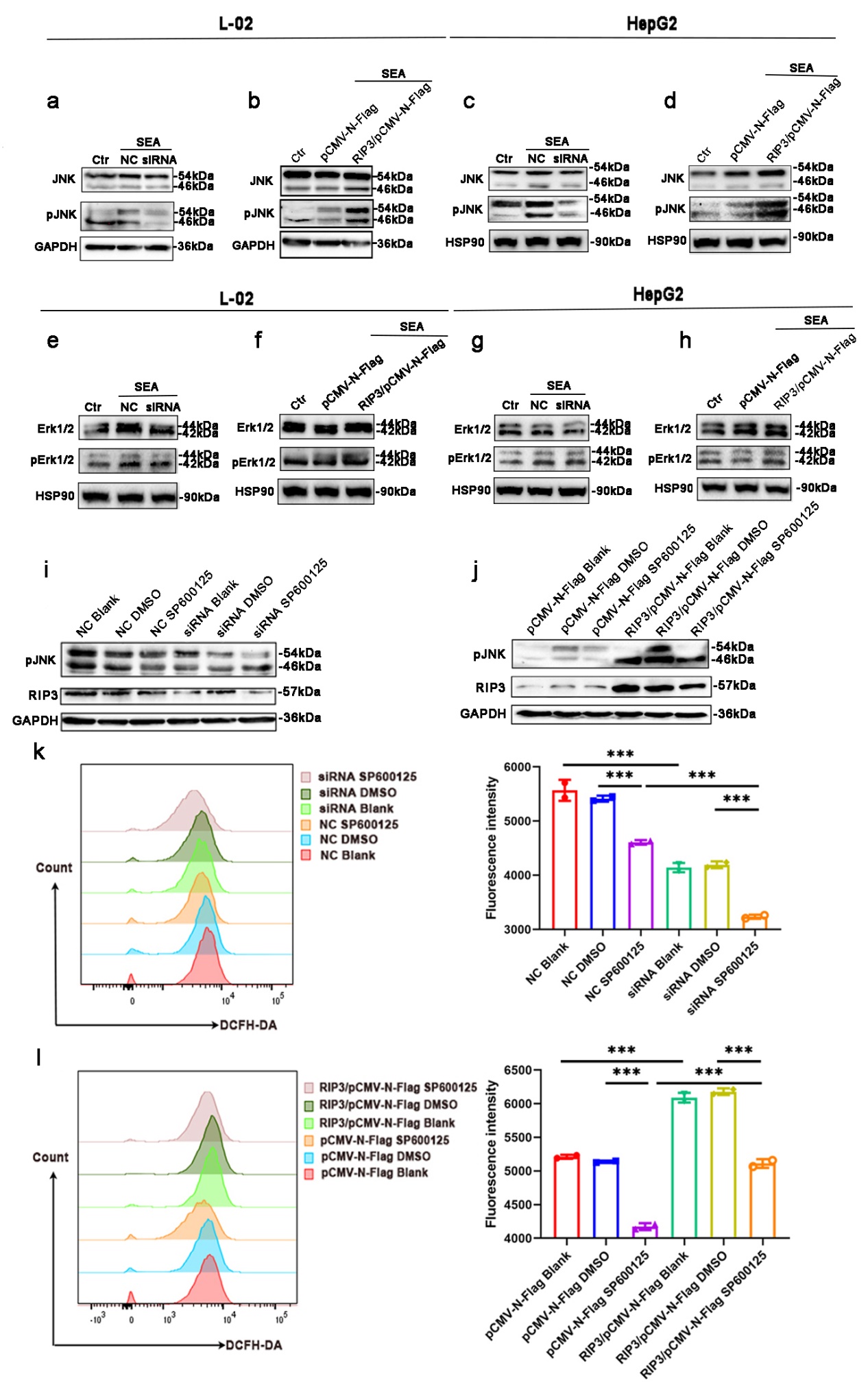


**Supplementary Fig. S6 The expressions of JNK, Erk1/2 and phosphorylation in L-02 or HepG2 cells.** **a.** The expressions of JNK and pJNK in L-02 cells transfected with RIP3 siRNA. **b.** The expression of JNK and pJNK in L-02 cells overexpressing RIP3. **c.** The expressions of JNK and pJNK in HepG2 cells transfected with RIP3 siRNA. **d**. The expression of JNK and pJNK in HepG2 cells overexpressing RIP3. **e**. The expressions of Erk1/2 and pErk1/2 in L-02 cells transfected with RIP3 siRNA. **f.** The expression of Erk1/2 and pErk1/2 in L-02 cells overexpressing RIP3. **g.** The expressions of Erk1/2 and pErk1/2 in HepG2 cells transfected with RIP3 siRNA. **h**. The expression of Erk1/2 and pErk1/2 in HepG2 cells overexpressing RIP3. **i.** The expression of RIP3 in SP600125-treated RIP3 knockdown L-02 cells. **j.** The expression of RIP3 in SP600125-treated RIP3-overexpressing L-02 cells. **k.** The fluorescence intensity of ROS in SP600125-treated RIP3 knockdown L-02 cells. **l.** The fluorescence intensity of ROS in SP600125-treated RIP3-overexpressing L-02 cells (**P* < 0.05, ***P* < 0.01, ****P* < 0.001).


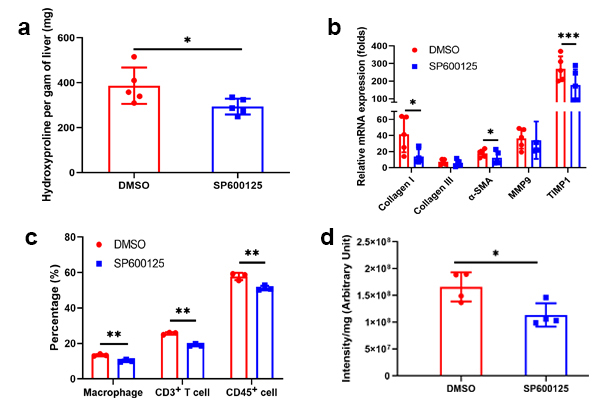


**Supplementary Fig. S7 Fibrosis and inflammatory levels** **in the livers of SP600125-treated infected mice. a.** Hydroxyproline levels in the livers of SP600125-treated infected mice. **b.** Collagen I, collagen III, α-SMA, MMP9, and TIMP1 mRNA levels in the livers of SP600125-treated infected mice detected by fluorescence quantitative PCR. **c.** Flow cytometry detection of the ratio of macrophages, CD3^+^ T cells, and CD45^+^ cells in the livers of SP600125-treated infected mice. **d.** ROS levels in the livers of SP600125-treated infected mice (**P* < 0.05, ***P* < 0.01, ****P* < 0.001).


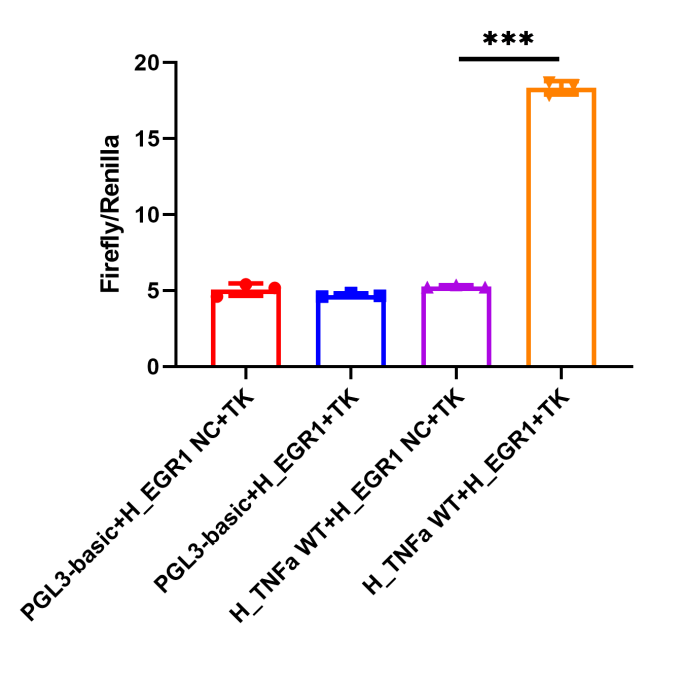


**Supplementary Fig. S8 Detection of Egr1 fluorescent reporter gene: Egr1 regulated the expression of TNF-α.** PGL3-basic + H_EGR1 NC + TK, promoter control plasmid + Egr1 control plasmid + Renilla luciferase reporter plasmid; PGL3-basic + H_EGR1 + TK, Promoter control plasmid + Egr1 overexpression plasmid + Renilla luciferase reporter plasmid; H_TNFa WT + H_EGR1 NC + TK, TNF- α Promoter plasmid + Egr1 control plasmid + Renilla luciferase reporter plasmid; H_TNFa WT + H_EGR1 + TK, TNF-α Promoter plasmid + Egr1 overexpression plasmid + Renilla luciferase reporter plasmid (****P* < 0.001).


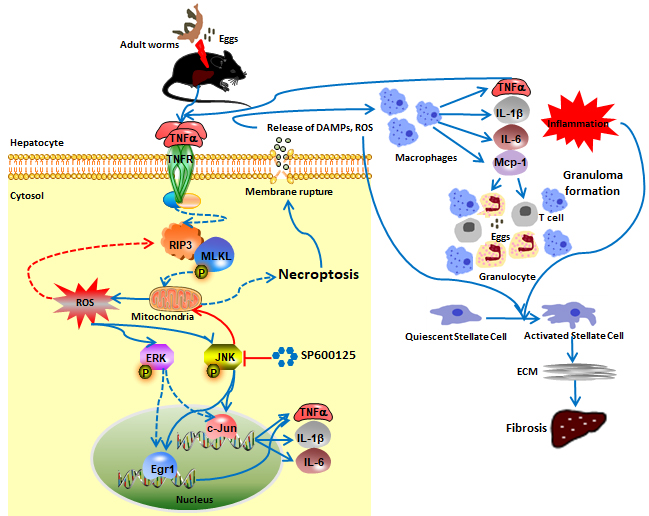


**Supplementary Fig. S9** **The proposed work model of RIP3 regulation liver fibrosis induced by *S. japonicum.***

**Supplementary Table S1 The sequences of the fluorescence quantitative PCR primers.**

| Genes | Primer | Sequence(5’–3’ ) |
| --- | --- | --- |
| 18S | Forward primer | TGCACCACCAACTGCTTAGC |
|  | Reverse primer | GTGGTCATGAGCCCTTCCA |
| Collagen I | Forward primer | GCGAGTGCTGTGCTTTCTG |
|  | Reverse primer | TCCCTCGACTCCTACATCTTC |
| Collagen III | Forward primer | CCCAACCCAGAGATCCCATT |
|  | Reverse primer | GAAGCACAGGAGCAGGTGTAGA |
| α-SMA | Forward primer | TCAGCGCCTCCAGTTCCT |
|  | Reverse primer | AAAAAAAACCACGAGTAACAAATCAA |
| MMP-9 | Forward primer | GCTCATGTACCCGCTGTATAGCT |
|  | Reverse primer | CAGATACTGGATGCCGTCTATGTC |
| TIMP-1 | Forward primer | TGGGAAATGCCGCAGATATC |
|  | Reverse primer | TGGGACTTGTGGGCATATCC |
| TNF-α | Forward primer | CCCTCACACTCAGATCATCTTCT |
|  | Reverse primer | GCTACGACGTGGGCTACAG |
| IL-1β | Forward primer | ATGGCAACTGTTCCTGAACTCAACT |
|  | Reverse primer | CAGGACAGGTATAGATTCTTTCCTTT |
| IL-6 | Forward primer | TAGTCCTTCCTACCCCAATTTCC |
|  | Reverse primer | TTGGTCCTTAGCCACTCCTTC |
| F4/80 | Forward primer | CCCCAGTGTCCTTACAGAGTG |
|  | Reverse primer | GTGCCCAGAGTGGATGTCT |
| MCP-1 | Forward primer | AGGTCCCTGTCATGCTTCTG |
|  | Reverse primer | TCTGGACCCATTCCTTCTTG |
| GPX4 | Forward primer | TTACGAATCCTGGCCTTCCC |
|  | Reverse primer | ACCACGCAGCCGTTCTTATC |
| ACSL4 | Forward primer | TGGAAGTCCATATCGCTCTGT |
|  | Reverse primer | TTGGCTACAGCATGGTCAAA |

**Supplementary Table S2 The RIP3 siRNA sequences.**

|  | Sense (5′–3) | Antisense (5′–3′) |
| --- | --- | --- |
| RIPK3-homo-775 | GAACUGUUUGUUAACGUAATT | UUACGUUAACAAACAGUUCTT |
| RIPK3-homo-478 | CCGGCUCUGGUGACUAAAUTT | AUUUAGUCACCAGAGCCGGTT |
| GAPDH Positive control | UGACCUCAACUACAUGGUUTT | AACCAUGUAGUUGAGGUCATT |
| Negative control | UUCUCCGAACGUGUCACGUTT | ACGUGACACGUUCGGAGAATT |
